# Relationship of *FTO* gene variations with NAFLD risk in Chinese men

**DOI:** 10.1515/biol-2020-0081

**Published:** 2020-11-30

**Authors:** Xuefen Chen, Yong Gao, Xiaobo Yang, Haiying Zhang, Zengnan Mo, Aihua Tan

**Affiliations:** Department of chemotherapy, The Affiliated Tumor Hospital of Guangxi Medical University, Nanning, Guangxi, 530021, China; Center for Genomic and Personalized Medicine, Guangxi Medical University, Nanning, Guangxi, 530021, China

**Keywords:** *FTO* gene, nonalcoholic fatty liver disease, SNPs, obesity

## Abstract

**Background:**

Fat mass and obesity-associated (*FTO*) gene is an obesity susceptibility gene and its relationship with the nonalcoholic fatty liver disease (NAFLD) remains unclear. This study aims to investigate the relationships of *FTO* gene variations with NAFLD risk in a Chinese male population.

**Methods:**

A 1:2 matched case–control study was performed on 275 cases of NAFLD and 550 controls matched for age. Nine of the *FTO* gene single nucleotide polymorphisms (SNPs) were genotyped.

**Results:**

Logistic regression analysis found that *FTO* rs1477196 was significantly associated with the susceptibility to NAFLD in recessive genetic models [unadjusted odds ratio (OR) = 2.52, 95% confidence interval (CI): 1.22–5.19, *P* = 0.012] and the relativity weakened after further adjustment for body mass index (BMI), uric acid, metabolic syndrome, smoking, and drinking (adjusted OR = 2.18, 95% CI: 0.96–4.99, *P* = 0.06). In the obese group, the AA + AG genotypes of rs1121980 and rs9940128 were associated with a decreased risk of NAFLD, when compared with the GG genotype, respectively (rs1121980: adjusted OR = 0.62, 95% CI = 0.39–0.99, *P* = 0.044; rs9940128: adjusted OR = 0.61, 95% CI = 0.38–0.97, *P* = 0.038). Furthermore, rs1477196 was associated with the severity of NAFLD (OR = 2.95, 95% CI = 1.09–7.94, *P* = 0.034).

**Conclusions:**

Our results demonstrated that the *FTO* gene was related to the presence and severity of NAFLD in a Chinese male population, and the relationships of the tested SNPs with NAFLD are most probably mediated by BMI.

## 1 Introduction

Nonalcoholic fatty liver disease (NAFLD), characterized by an excessive fat deposition in hepatocytes, excluding alcohol and other specific liver damaging factors [1], is an acquired metabolic stress liver injury closely related to insulin resistance and genetic predisposition. It can not only directly lead to cirrhosis and hepatocellular carcinoma but also affect the progression of other chronic liver diseases and be involved in the pathogenesis of type 2 diabetes and atherosclerosis [2]. It is estimated that NAFLD will become the leading cause of liver-related morbidity and mortality within 20 years.

The fat mass and obesity-associated (*FTO*) gene is located on chromosome 16q12.2. As a predictor of metabolic disorders, the *FTO* gene plays a conclusive role in the command of energy balance and is highly expressed in many tissues, including fat and liver [3,4]. The relationships of *FTO* gene polymorphisms with metabolic diseases have been extensively studied. *FTO* rs9939609 and rs17817449 were reported to be related to metabolic syndrome, type 2 diabetes mellitus, and obesity [5,6]; rs8050136 and rs7195539 were associated with type 2 diabetes mellitus in a Uyghur population [7]. A study in western Spain found that rs9921255 and rs1477196 could increase the risk of obesity-related traits [8]. Other studies found that rs1121980 and rs8061518 were strongly related to obesity [9,10], and rs9940128 had relationships with type 2 diabetes mellitus and obesity in south Indians [11]. Besides, Haupt et al. [12] have reported the relationship of *FTO* gene polymorphism with liver fat content and found that there was a significant effect of *FTO* rs8050136 on subcutaneous fat and a trend for liver fat content.

NAFLD is closely related to metabolic disorders, such as insulin resistance and obesity [13], and is also related to variability in some important NAFLD genes (i.e., *PNPLA3* and *TM6SF2*) [14]. So far, the relationships of *FTO* gene variants with NAFLD risk remain unclear. Our study was designed to explore the relationships of *FTO* gene variations with NAFLD risk in a Chinese male population.

## 2 Participants and methods

### 2.1 Study population

We used a 1:2 nested case–control study design in our study, in which one NAFLD patient was matched to two non-NAFLD men on age (±3 years). The age-matched controls were selected randomly from all subjects without NAFLD. All participants were from the FAMHES cohort [15], which included 4,303 continuous male health examinees in the Medical Centre of Fangchenggang First People’s Hospital from September 2009 to December 2009. And participants with the following criteria were excluded: (1) coronary heart disease, stroke, diabetes mellitus, hyperthyroidism, or cancer; (2) hepatitis history; (3) heavy drinkers (≥ 20 g/day, according to the published report [16]); and (4) without ultrasound diagnostic data. A total of 334 men were diagnosed with NAFLD and 59 of them had no data on genotyping. In the end, 275 cases of NAFLD and 550 controls matched for age in 1:2 were included in the analysis.


**Informed consent:** Informed consent has been obtained from all individuals included in this study.
**Ethical approval:** The research related to human use has been complied with all the relevant national regulations, institutional policies and in accordance with the tenets of the Helsinki Declaration and has been approved by the Guangxi Medical University Ethics Committee.

### 2.2 Data collection

We collected participants’ age, smoking status, alcohol consumption, physical activity, and medical history by the questionnaire survey methods. Drinkers were defined as consuming at least one drink of alcohol (beer, wine, or hard liquor) per week. Smokers were defined as smoking at least once a day for more than 6 months. The exercise intensity was classified as low, moderate, or high according to the questionnaire scoring protocol [15]. We measured the height, body weight, waist circumference (WC), and blood pressure (BP) according to a standard protocol. Body mass index (BMI) was calculated as weight (kg)/[height (m)]^2^, with BMI < 25.0 defined as normal weight and BMI ≥ 25 as obese [17]. Metabolic syndrome was defined as including the following three or more components [15]: (1) WC ≥ 90 cm; (2) triglycerides (TG) ≥ 1.7 mmol/L; (3) high-density lipoprotein cholesterol (HDL-c) < 1.03 mmol/L; (4) BP ≥ 130/85 mmHg or current use of antihypertensive medications; and (5) fasting blood glucose (FBG) ≥ 5.6 mmol/L.

### 2.3 Definition of NAFLD

The NAFLD was diagnosed with abdominal ultrasound, excluding the other causes [excessive drinking (≥ 20 g/day), viral or autoimmune liver disease, etc.] of chronic liver disease [18]. The liver size, structure, contour, echogenicity, and posterior beam attenuation were assessed independently by two sonographers using a portable ultrasound device (GE LOGIQ e, 5.0 MHz transducer; GE Healthcare, Wauwatosa, Wisconsin, USA). Participants were ultrasonically diagnosed of fatty liver when having the following two or three symptoms [19]: (1) diffused liver enhanced near-field echo, with an echo intensity higher than that of the kidney; (2) dirty liver far-field echo decays; and (3) intrahepatic duct structure display is unclear.

### 2.4 Genotyping

The venous blood samples were collected, and genomic DNA was extracted. Nine single nucleotide polymorphisms (SNPs) (rs9939609, rs1121980, rs17817449, rs8050136, rs9940128, rs8061518, rs9921255, rs1477196, and rs7195539) of the *FTO* gene were selected, and these SNPs were reported to be related to metabolic disorders such as obesity, metabolic syndrome, and type 2 diabetes [5,6,7,8,9,10,11]. The genotyping method has been described previously [20]. All genotyping reactions were performed in 384-well plates, and each plate included a duplicate and a negative control for 3–4 samples selected at random. The average concordance rate was 99.8%.

### 2.5 Statistical analysis

Numeric variables were described as mean ± standard deviation (SD) or median (quartile range) and analyzed with the *t*-test or rank-sum test. Categorical data were described as percentages (%) and analyzed using the *x* ^2^ test. Hardy–Weinberg equilibrium (HWE) was computed with the *x* ^2^ test to compare the observed genotype frequencies with the expected genotype frequencies among the controls. We performed the binary logistic regression analysis to calculate the odds ratio (OR) and 95% confidence intervals (CIs) and evaluate the relationships of SNPs with NAFLD risk. The confounding factors included BMI, uric acid, metabolic syndrome, smoking, and drinking. All statistical analyses were performed using SPSS 17.0 (Chicago, IL, USA) and SNPStats (a web tool for the analysis of association studies) [21], and *P* < 0.05 was considered statistically significant.

## 3 Results

Characteristics of the 275 cases of NAFLD and 550 controls are described in Table 1. The mean age of the NAFLD group was 39.26 ± 1.28 years, similar to the controls (39.23 ± 1.28 years, *P* = 0.958). As expected, compared with the controls, the prevalence of metabolic syndrome and the levels of FBG, alanine aminotransferase (ALT), uric acid, TG, total cholesterol (TC), and low-density lipoprotein cholesterol (LDL-c) were all higher (all *P* < 0.01), and the HDL-c level was lower in the NAFLD group (*P* < 0.001). Besides, there were more smokers and drinkers among NAFLD patients (all *P* < 0.001). However, there was no difference in physical activity between the two groups (*P* = 0.539). Among the 275 NAFLD patients, the mild, moderate, and severe steatosis were 195 (70.91%), 62 (22.55%), and 18 (6.55%), respectively.

**Table 1:** Baseline characteristics of the study population stratified for the absence and presence of NAFLD

Characteristic	NAFLD group	Control group	*P*
*n*	275	550	
Age (years)	39.26 ± 1.28	39.23 ± 1.28	0.958
BMI	26.40 ± 2.81	23.11 ± 3.07	<0.001
Glucose (mmol/L)	5.51 ± 1.20	5.28 ± 1.14	0.001
ALT (mmol/L)	47.00 ± 1.44	41.13 ± 1.60	<0.001
TC (mmol/L)	6.06 ± 1.04	5.73 ± 1.05	<0.001
TG (mmol/L)	1.95 ± 1.85	1.18 ± 1.87	<0.001
HDL (mmol/L)	1.26 ± 1.28	1.38 ± 1.23	<0.001
LDL (mmol/L)	3.24 ± 0.78	2.98 ± 0.83	<0.001
Uric acid (µmol/L)	412.34 ± 74.88	369.52 ± 81.42	<0.001
Metabolic syndrome (*n*, %)	72 (26.18)	42 (7.64)	<0.001
DBP (mmHg) (M, QR)	80 (18)	78 (10)	<0.001
SBP (mmHg) (M, QR)	120 (20)	120 (18.5)	0.002
Smoking (*n*, %)	227 (82.55)	304 (55.27)	<0.001
Drinking (*n*, %)	149 (54.18)	83 (15.10)	<0.001
Physical activity (*n*, %)			0.539
Low	178 (64.7)	368 (66.9)	
Moderate	71 (25.8)	139 (25.3)	
High	26 (9.5)	40 (7.3)	
The severity of NAFLD (*n*, %)			—
Mild	195 (70.91)	—	
Moderate	62 (22.55)	—	
Severe	18 (6.55)	—	

Data are presented as mean ± SD. *n*, number; BMI, body mass index; ALT, alanine aminotransferase; TC, total cholesterol; TG, triglycerides; HDL, high-density lipoprotein; LDL, low-density lipoprotein; DBP, diastolic blood pressure; SBP, systolic blood pressure; M, median; QR, quartile range.

The genotype frequencies of the nine selected SNPs and their associations with risk of NAFLD are shown in Table 2. Logistic regression analysis showed that rs1477196 was significantly associated with the susceptibility to NAFLD in recessive genetic models (model 1), and carriers of the AA genotype increased the NAFLD risk in comparison with AG + GG carriers (OR = 2.52, 95% CI: 1.22–5.19, *P* = 0.012). However, the relativity weakened after adjustment for BMI (OR = 2.10, 95% CI: 0.93–4.72, *P* = 0.07 in model 2) and further for uric acid, metabolic syndrome, smoking, and drinking (OR = 2.18, 95% CI: 0.96–4.99, *P* = 0.06 in model 3). The other SNPs were not associated with the NAFLD risk in all genetic models. Besides, all the nine SNPs in the control group were in HWE (all *P* > 0.05, data not shown).

**Table 2:** Distribution of the genotypes of *FTO* and their associations with risk of NAFLD

		Genotype frequencies, *N*		Model 1		Model 2		Model 3	
		NAFLD	Controls	OR (95% CI)	*P*	OR (95% CI)	*P*	OR (95% CI)	*P*
rs1121980									
Dominant	(AA + AG)/GG	88/187	192/357	0.88 (0.64–1.19)	0.39	0.79 (0.56–1.12)	0.18	0.80 (0.56–1.14)	0.21
Recessive	AA/(AG + GG)	14/261	16/533	1.79 (0.86–3.72)	0.12	1.68 (0.73–3.87)	0.22	1.64 (0.70–3.81)	0.26
Additive	AA/AG/GG	14/74/187	16/176/357	0.97 (0.75–1.27)	0.85	0.90 (0.67–1.21)	0.48	0.90 (0.67–1.22)	0.50
rs1477196									
Dominant	(AA + AG)/GG	94/180	194/353	0.95(0.70–1.29)	0.74	0.95 (0.67–1.34)	0.77	0.93 (0.66–1.32)	0.70
Recessive	AA/(AG + GG)	17/257	14/533	2.52 (1.22–5.19)	0.012	2.10 (0.93–4.72)	0.07	2.18 (0.96–4.99)	0.06
Additive	AA/AG/GG	17/77/180	14/180/353	1.08 (0.84–1.40)	0.55	1.06 (0.79–1.41)	0.69	1.05 (0.79–1.41)	0.73
rs17817449									
Dominant	(GG + TG)/TT	68/207	144/406	0.93 (0.66–1.29)	0.65	0.78 (0.53–1.13)	0.18	0.78 (0.53–1.15)	0.20
Recessive	GG/(TG + TT)	8/267	9/541	1.80 (0.69–4.72)	0.24	1.34 (0.45–3.98)	0.60	1.26 (0.41–3.87)	0.69
Additive	GG/TG/TT	8/60/207	9/135/406	0.99 (0.74–1.33)	0.96	0.84 (0.60–1.18)	0.31	0.84 (0.60–1.18)	0.32
rs7195539									
Dominant	(AG + GG)/AA	46/228	111/437	0.79 (0.54–1.16)	0.23	0.75 (0.49–1.15)	0.18	0.72 (0.47–1.11)	0.13
Recessive	GG/(AA + AG)	4/270	7/541	1.14 (0.33–3.95)	0.83	2.38 (0.60–9.42)	0.23	2.48 (0.61–10.05)	0.21
Additive	GG/AG/AA	4/42/228	7/104/437	0.84 (0.59–1.18)	0.30	0.83 (0.56–1.24)	0.36	0.81 (0.54–1.21)	0.30
rs8050136									
Dominant	(AA + AC)/CC	68/207	142/407	0.94 (0.67–1.31)	0.72	0.79 (0.54–1.16)	0.22	0.80 (0.55–1.18)	0.25
Recessive	AA/(AC + CC)	8/267	8/541	2.03 (0.75–5.46)	0.17	1.54 (0.50–4.77)	0.46	1.48 (0.46–4.73)	0.51
Additive	AA/AC/CC	8/60/207	8/134/407	1.01 (0.75–1.36)	0.93	0.86 (0.62–1.21)	0.39	0.87 (0.62–1.22)	0.41
rs8061518									
Dominant	(AG + GG)/AA	183/92	342/206	1.20 (0.88–1.62)	0.24	1.11 (0.79–1.57)	0.54	1.14 (0.80–1.61)	0.47
Recessive	GG/(AA + AG)	57/218	91/457	1.31 (0.91–1.90)	0.15	1.28 (0.85–1.94)	0.24	1.33 (0.87–2.02)	0.19
Additive	GG/AG/AA	57/126/92	91/251/206	1.18 (0.96–1.44)	0.12	1.13 (0.90–1.42)	0.29	1.15 (0.91–1.45)	0.23
rs9921255									
Dominant	(TC + CC)/TT	53/215	107/421	0.97 (0.67–1.40)	0.87	0.99 (0.65–1.49)	0.95	1.03 (0.68–1.57)	0.88
Recessive	CC/(TC + TT)	3/265	4/524	1.48 (0.33–6.67)	0.61	1.00 (0.18–5.47)	1.00	1.04 (0.19–5.80)	0.96
Additive	CC/TC/TT	3/50/215	4/103/421	0.99 (0.70–1.40)	0.97	0.99 (0.67–1.45)	0.95	1.03 (0.70–1.52)	0.88
rs9939609									
Dominant	(AA + TA)/TT	68/207	140/406	0.95 (0.68–1.32)	0.74	0.79 (0.54–1.16)	0.23	0.80 (0.55–1.18)	0.26
Recessive	AA/(TT + TA)	8/267	8/539	2.02 (0.75–5.44)	0.17	1.53 (0.49–4.74)	0.46	1.46 (0.46–4.69)	0.52
Additive	AA/TA/TT	8/60/207	7/133/406	1.02 (0.76–1.37)	0.91	0.87 (0.62–1.21)	0.40	0.87 (0.62–1.22)	0.42
rs9940128									
Dominant	(AA + AG)/GG	89/186	195/355	0.87 (0.64–1.18)	0.38	0.77 (0.54–1.09)	0.14	0.78 (0.55–1.10)	0.16
Recessive	AA/(GG + AG)	14/261	17/533	1.68 (0.82–3.46)	0.16	1.66 (0.73–3.79)	0.23	1.60 (0.69–3.72)	0.27
Additive	AA/AG/GG	14/75/186	17/178/355	0.97 (0.74–1.25)	0.79	0.88 (0.66–1.18)	0.40	0.88 (0.65–1.19)	0.41

Model 1 is not adjusted for other factors; model 2 is adjusted for BMI; model 3 is adjusted for BMI, uric acid, metabolic syndrome, smoking, and drinking.

We further evaluated the effect of *FTO* gene polymorphisms on NAFLD risk stratified by BMI. When BMI ≥ 25, significant correlations were found between genotypes of rs1121980, rs9940128 and susceptibility to NAFLD (Table 3). The AA + AG genotypes of rs1121980 and rs9940128 were associated with a decreased risk of NAFLD, compared with the GG genotype, respectively (rs1121980: adjusted OR = 0.62, 95% CI = 0.39–0.99, *P* = 0.044; rs9940128: adjusted OR = 0.61, 95% CI = 0.38–0.97, *P* = 0.038). No significant correlations were observed between the nine SNPs of *FTO* and NAFLD risk in all genetic models when BMI < 25 (Table S1).

**Table 3:** Distribution of the genotypes of *FTO* and their associations with risk of NAFLD when BMI ≥ 25

	Genotype distribution, *N* (%)		Dominant model		Recessive model		Additive model	
	NAFLD	Controls	OR^a^ (95% CI)	*P*	OR^a^ (95% CI)	*P*	OR^a^ (95% CI)	*P*
rs1121980			0.62 (0.39–0.99)	0.044	4.07 (0.88–18.83)	0.14	0.81 (0.55–1.21)	0.31
GG	133 (69.3)	78 (59.1)						
AG	47 (24.5)	52 (39.4)						
AA	12 (6.2)	2 (1.5)						
rs1477196			0.71 (0.44–1.14)	0.16	2.06 (0.72–5.90)	0.16	0.90 (0.62–1.30)	0.56
GG	130 (68.1)	80 (60.6)						
AG	47 (24.6)	47 (35.6)						
AA	14 (7.3)	5 (3.8)						
rs17817449			0.67 (0.41–1.11)	0.12	2.15 (0.43–10.81)	0.33	0.79 (0.51–1.22)	0.29
TT	144 (75)	89 (67.4)						
GT	41 (21.4)	41 (31.1)						
GG	7 (3.6)	2 (1.5)						
rs7195539			0.90 (0.51–1.59)	0.71	—	—	1.00 (0.59–1.71)	0.99
AA	156 (81.7)	106 (80.3)						
GA	32 (16.8)	26 (19.7)						
GG	3 (1.6)	0 (0)						
rs8050136			0.70 (0.43–1.15)	0.16	2.15 (0.43–10.81)	0.33	0.82 (0.53–1.26)	0.36
CC	144 (75)	144 (75)						
AC	41 (21.4)	41 (21.4)						
AA	7 (3.6)	2 (1.5)						
rs8061518			1.21 (0.76–1.95)	0.42	1.35 (0.77–2.37)	0.29	1.19 (0.87–1.62)	0.27
AA	63 (32.8)	49 (37.4)						
GA	85 (44.3)	58 (44.3)						
GG	44 (22.9)	24 (18.3)						
rs9921255			1.30 (0.71–2.36)	0.39	1.91 (0.19–19.55)	0.57	1.29 (0.75–2.24)	0.35
TT	150 (80.2)	106 (83.5)						
CT	34 (18.2)	20 (15.8)						
CC	3 (1.6)	3 (1.6)						
rs9939609			0.69 (0.42–1.14)	0.15	2.12 (0.42–10.66)	0.34	0.81 (0.52–1.24)	0.33
TT	144 (75)	89 (67.9)						
AT	41 (21.4)	40 (30.5)						
AA	7 (3.6)	2 (1.5)						
rs9940128			0.61 (0.38–0.97)	0.038	4.07 (0.88–18.83)	0.14	0.81 (0.54–1.19)	0.28
GG	132 (68.8)	77 (58.3)						
AG	48 (25)	53 (40.1)						
AA	12 (6.2)	2 (1.5)						

a Adjusted for BMI, uric acid, metabolic syndrome, smoking, and drinking.

**Table S1:** Distribution of the genotypes of *FTO* and their associations with risk of NAFLD when BMI < 25

	Genotype distribution, *N* (%)		Dominant model		Recessive model		Additive model	
	NAFLD	Controls	OR^a^ (95% CI)	*P*	OR^a^ (95% CI)	*P*	OR^a^ (95% CI)	*P*
rs1121980			0.98 (0.57–1.69)	0.95	0.62 (0.13–2.97)	0.53	0.94 (0.59–1.50)	0.79
GG	53 (64.6)	279 (66.9)						
AG	27 (32.9)	124 (29.7)						
AA	2 (2.4)	14 (3.4)						
rs1477196			1.38 (0.81–2.33)	0.24	2.39 (0.57–10.10)	0.26	1.40 (0.88–2.24)	0.16
GG	49 (59.8)	273 (65.8)						
AG	30 (36.6)	133 (32)						
AA	3 (3.7)	9 (2.2)						
rs17817449			0.81 (0.44–1.49)	0.50	0.49 (0.05–4.44)	0.49	0.80 (0.46–1.39)	0.43
TT	62 (75.6)	317 (75.8)						
GT	19 (23.2)	94 (22.5)						
GG	1 (1.2)	7 (1.7)						
rs7195539			0.59 (0.29–1.23)	0.14	1.41 (0.15–13.56)	0.77	0.66 (0.33–1.29)	0.20
AA	71 (86.6)	331 (79.6)						
GA	10 (12.2)	78 (18.8)						
GG	1 (1.2)	7 (1.7)						
rs8050136			0.81 (0.44–1.49)	0.50	0.74 (0.08–6.90)	0.78	0.82 (0.47–1.44)	0.49
CC	62 (75.6)	317 (76.0)						
AC	19 (23.2)	94 (22.5)						
AA	1 (1.2)	6 (1.4)						
rs8061518			1.16 (0.67–2.02)	0.59	1.19 (0.59–2.39)	0.62	1.13 (0.77–1.66)	0.53
AA	29 (35.4)	157 (37.6)						
GA	40 (48.8)	193 (46.3)						
GG	13 (15.8)	67 (16.1)						
rs9921255			0.81 (0.42–1.55)	0.53	–	–	0.79 (0.42–1.48)	0.45
TT	65 (81.2)	315 (78.5)						
CT	15 (18.8)	83 (20.7)						
CC	0 (0)	3 (0.8)						
rs9939609			0.84 (0.46–1.54)	0.57	0.73 (0.08–6.82)	0.77	0.84 (0.48–1.48)	0.55
TT	62 (75.6)	317 (76.2)						
AT	19 (23.2)	93 (22.4)						
AA	1 (1.2)	6 (1.4)						
rs9940128			0.97 (0.57–1.67)	0.92	0.61 (0.13–2.92)	0.51	0.93 (0.58–1.49)	0.76
GG	53 (64.6)	278 (66.5)						
AG	27 (32.9)	125 (29.9)						
AA	2 (2.4)	15 (3.6)						

a Adjusted for BMI, uric acid, metabolic syndrome, smoking, and drinking.

Linkage disequilibrium information on the nine SNPs is shown in Table S2. Haplotype analysis of nine SNPs in *FTO* was performed to evaluate the effect of haplotypes on NAFLD risk, and no significant relationships were found in all subjects or those with BMI ≥ 25 or < 25 (Tables S3 and S4). The analysis of the associations between the nine SNPs and BMI is reported in Table S5, and no associations were found in the subject with NAFLD or the controls (all *P* > 0.05).

**Table S2:** Linkage disequilibrium between pairs of the nine SNPs

	rs1477196	rs17817449	rs7195539	rs8050136	rs8061518	rs9921255	rs9939609	rs9940128
rs1121980	*D* = 0.998	*D* = 1.000	*D* = 0.325	*D* = 1.000	*D* = 0.134	*D* = 0.018	*D* = 1.000	*D* = 1.000
	*r* ^2^ = 0.056	*r* ^2^ = 0.697	*r* ^2^ = 0.052	*r* ^2^ = 0.686	*r* ^2^ = 0.003	*r* ^2^ = 0.000	*r* ^2^ = 0.693	*r* ^2^ = 0.980
rs1477196		*D* = 0.994	*D* = 0.356	*D* = 0.994	*D* = 0.279	*D* = 0.033	*D* = 0.994	*D* = 0.998
		*r* ^2^ = 0.039	*r* ^2^ = 0.003	*r* ^2^ = 0.038	*r* ^2^ = 0.027	*r* ^2^ = 0.000	*r* ^2^ = 0.038	*r* ^2^ = 0.057
rs17817449			*D* = 0.144	*D* = 1.000	*D* = 0.041	*D* = 0.124	*D* = 1.000	*D* = 1.000
			*r* ^2^ = 0.015	*r* ^2^ = 0.985	*r* ^2^ = 0.000	*r* ^2^ = 0.000	*r* ^2^ = 1.000	*r* ^2^ = 0.683
rs7195539				*D* = 0.147	*D* = 0.999	*D* = 0.103	*D* = 0.140	*D* = 0.320
				*r* ^2^ = 0.015	*r* ^2^ = 0.079	*r* ^2^ = 0.010	*r* ^2^ = 0.014	*r* ^2^ = 0.049
rs8050136					*D* = 0.038	*D* = 0.100	*D* = 1.000	*D* = 1.000
					*r* ^2^ = 0.000	*r* ^2^ = 0.000	*r* ^2^ = 1.000	*r* ^2^ = 0.673
rs8061518						*D* = 0.063	*D* = 0.045	*D* = 0.133
						*r* ^2^ = 0.000	*r* ^2^ = 0.000	*r* ^2^ = 0.003
rs9921255							*D* = 0.096	*D* = 0.051
							*r* ^2^ = 0.000	*r* ^2^ = 0.000
rs9939609								*D* = 1.000
								*r* ^2^ = 0.680

**Table S3:** Association analysis of haplotypes derived from polymorphic sites using genotype data.

Haplotype	1	2	3	4	5	6	7	8	9	OR (95% CI)	*P*1	OR (95% CI)	*P*2	OR (95% CI)	*P*3
H1	G	G	T	A	C	G	T	T	G	0.76 (0.55–1.06)	0.11	0.84 (0.58–1.22)	0.36	0.86 (0.58–1.26)	0.43
H2	G	A	T	A	C	G	T	T	G	0.88 (0.59–1.30)	0.52	0.93 (0.60–1.45)	0.75	0.88 (0.56–1.39)	0.58
H3	G	A	T	A	C	A	T	T	G	0.98 (0.56–1.71)	0.93	1.21 (0.63–2.33)	0.56	1.32 (0.67–2.59)	0.43
H4	A	G	G	A	A	G	T	A	A	0.97 (0.55–1.72)	0.92	1.22 (0.60–2.48)	0.59	1.16 (0.57–2.39)	0.68
H5	A	G	G	A	A	A	T	A	A	0.85 (0.46–1.58)	0.62	0.99 (0.47–2.09)	0.98	1.04 (0.47–2.28)	0.92
H6	G	G	T	G	C	A	T	T	G	2.37 (0.90–6.23)	0.08	2.64 (0.86–8.14)	0.09	2.43 (0.78–7.56)	0.13
H7	G	G	T	A	C	G	C	T	G	1.21 (0.45–3.29)	0.71	1.20 (0.43–3.34)	0.73	1.13 (0.40–3.19)	0.82
H8	G	G	T	A	C	A	C	T	G	0.75 (0.26–2.19)	0.60	0.77 (0.23–2.61)	0.68	0.76 (0.21–2.72)	0.67
H9	A	G	G	G	A	A	T	A	A	0.94 (0.42–2.12)	0.88	1.16 (0.44–3.08)	0.77	1.21 (0.45–3.27)	0.70
H10	A	G	T	G	C	A	T	T	A	0.67 (0.30–1.52)	0.34	0.69 (0.28–1.72)	0.43	0.68 (0.27–1.74)	0.42
H11	A	G	T	A	C	A	T	T	A	1.75 (0.59–5.16)	0.31	1.31 (0.40–4.29)	0.65	1.34 (0.40–4.52)	0.64
H12	G	G	T	G	C	A	C	T	G	1.06 (0.33–3.40)	0.92	1.29 (0.37–4.50)	0.69	1.35 (0.39–4.64)	0.22
H13	G	A	T	G	C	A	T	T	G	0.51 (0.13–2.03)	0.34	0.37 (0.08–1.62)	0.19	0.40 (0.09–1.72)	1.00

1, rs1121980; 2, rs1477196; 3, rs17817449; 4, rs7195539; 5, rs8050136; 6, rs8061518; 7, rs9921255; 8, rs9939609; 9, rs9940128; *P*1 value is not adjusted for other factors; *P*2 value is adjusted for BMI; *P*3 is adjusted for BMI, uric acid, metabolic syndrome, smoking, and drinking.

**Table S4:** Association analysis of haplotypes derived from polymorphic sites using genotype data stratified by BMI

Haplotype	1	2	3	4	5	6	7	8	9	OR (95% CI)	*P*1	OR (95% CI)	*P*2	OR (95% CI)	*P*3
BMI ≥ 25															
H1	G	G	T	A	C	G	T	T	G	0.69 (0.42–1.13)	0.14	0.69 (0.42–1.14)	0.14	0.69 (0.41–1.16)	0.16
H2	G	A	T	A	C	G	T	T	G	1.17 (0.65–2.09)	0.60	1.17 (0.65–2.10)	0.61	1.14 (0.63–2.04)	0.67
H3	G	A	T	A	C	A	T	T	G	1.03 (0.50–2.13)	0.93	1.04 (0.50––2.17)	0.91	1.03 (0.50–2.14)	0.93
H4	A	G	G	A	A	A	T	A	A	0.76 (0.33–1.76)	0.53	0.78 (0.33–1.83)	0.56	0.73 (0.30–1.74)	0.47
H5	A	G	G	A	A	G	T	A	A	1.26 (0.57–2.79)	0.57	1.25 (0.55–2.82)	0.60	1.40 (0.61–3.20)	0.43
H6	G	G	T	A	C	A	C	T	G	1.25 (0.32–4.84)	0.74	1.22 (0.32–4.68)	0.77	1.23 (0.34–4.41)	0.75
H7	A	G	G	G	A	A	T	A	A	1.34 (0.36–4.96)	0.66	1.39 (0.37–5.18)	0.63	1.18 (0.31–4.46)	0.81
H8	G	G	T	G	C	A	T	T	G	0.52 (0.15–1.82)	0.30	0.49 (0.14–1.75)	0.27	0.54 (0.15–1.88)	0.33
H9	A	G	T	G	C	A	T	T	A	0.77 (0.19–3.06)	0.71	0.83 (0.20–3.39)	0.80	0.87 (0.21–3.55)	0.85
H10	G	G	T	A	C	G	C	T	G	0.66 (0.16–2.77)	0.57	0.64 (0.15–2.75)	0.55	0.62 (0.15–2.61)	0.51
BMI < 25															
H1	G	G	T	A	C	G	T	T	G	1.11 (0.62–1.98)	0.73	1.11 (0.60–2.06)	0.73	0.98 (0.51–1.89)	0.96
H2	G	A	T	A	C	G	T	T	G	0.65 (0.36–1.17)	0.15	0.70 (0.37–1.31)	0.26	0.55 (0.28–1.06)	0.08
H3	A	G	G	A	A	G	T	A	A	1.53 (0.47–4.98)	0.48	1.58 (0.52–4.80)	0.42	1.45 (0.46–4.53)	0.52
H4	G	A	T	A	C	A	T	T	G	1.27 (0.37–4.34)	0.70	1.17 (0.34–4.05)	0.81	1.42 (0.40–5.05)	0.59
H5	A	G	G	A	A	A	T	A	A	0.60 (0.19–1.84)	0.37	0.79 (0.25–2.54)	0.70	0.72 (0.20–2.54)	0.61
H6	G	G	T	G	C	A	T	T	G	3.09 (0.44–22.01)	0.26	2.57 (0.28–23.43)	0.40	2.63 (0.29–24.27)	0.39
H7	G	G	T	A	C	G	C	T	G	0.55 (0.17–1.83)	0.33	0.67 (0.19–2.39)	0.53	2.21 (0.36–13.58)	0.39
H8	G	G	T	A	C	A	C	T	G	1.92 (0.36–10.34)	0.45	2.46 (0.43–13.97)	0.31	0.52 (0.14–1.97)	0.33
H9	A	G	T	A	C	A	T	T	A	1.05 (0.29–3.84)	0.94	0.92 (0.24–3.57)	0.90	0.86 (0.22–3.44)	0.84
H10	A	G	G	G	A	A	T	A	A	0.96 (0.24–3.95)	0.96	1.07 (0.26–4.32)	0.93	1.20 (0.28–5.06)	0.81
H11	A	G	T	G	C	A	T	T	A	1.34 (0.22–8.17)	0.75	1.48 (0.22–9.74)	0.69	0.98 (0.16–6.21)	0.98
H12	G	A	T	G	C	A	T	T	G	0.59 (0.10–3.36)	0.55	0.44 (0.05–3.50)	0.44	0.43 (0.06–3.25)	0.41
H13	A	G	T	A	C	G	T	T	A	0.29 (0.04–1.87)	0.19	0.37 (0.06–2.41)	0.30	0.24 (0.04–1.58)	0.14

1, rs1121980; 2, rs1477196; 3, rs17817449; 4, rs7195539; 5, rs8050136; 6, rs8061518; 7, rs9921255; 8, rs9939609; 9, rs9940128; *P*1 value is not adjusted for other factors; *P*2 value is adjusted for BMI; *P*3 is adjusted for BMI, uric acid, metabolic syndrome, smoking, and drinking.

**Table S5:** Association between SNPs and BMI investigated in NAFLD and control groups

		NAFLD group			Control group		
		*n*	M ± SD	*P*	*n*	M ± SD	*P*
rs1121980	GG	180	26.47 ± 2.862	0.45	357	22.43 ± 2.982	0.14
	AG	77	26.10 ± 2.830		176	22.97 ± 3.348	
	AA	17	26.94 ± 2.221		16	22.13 ± 3.096	
rs1477196	GG	187	26.43 ± 2.848	0.53	353	22.57 ± 3.069	0.95
	GA	74	26.18 ± 2.785		180	22.66 ± 3.238	
	AA	14	27.07 ± 2.515		14	22.64 ± 2.818	
rs17817449	TT	207	26.34 ± 2.859	0.55	406	22.43 ± 2.995	0.16
	GT	60	26.43 ± 2.697		135	22.43 ± 3.406	
	GG	8	27.45 ± 2.534		9	22.89 ± 3.371	
rs7195539	AA	228	26.33 ± 2.832	0.36	437	22.59 ± 3.110	0.07
	GA	42	26.87 ± 2.807		104	22.80 ± 3.120	
	GG	4	25.19 ± 1.571		7	20.00 ± 2.380	
rs8050136	CC	207	26.34 ± 2.859	0.55	407	22.44 ± 3.003	0.16
	AC	60	26.43 ± 2.697		134	23.04 ± 3.375	
	AA	8	27.45 ± 2.534		8	22.75 ± 3.576	
rs8061518	AA	92	26.36 ± 3.138	0.94	206	22.42 ± 3.001	0.60
	GA	126	26.37 ± 2.681		251	22.71 ± 3.265	
	GG	57	26.52 ± 2.578		91	22.62 ± 2.947	
rs9921255	TT	215	26.30 ± 2.846	0.58	421	22.63 ± 3.198	0.73
	CT	50	26.59 ± 2.421		103	22.40 ± 2.795	
	CC	3	27.66 ± 2.346		4	23.25 ± 3.304	
rs9939609	TT	207	26.34 ± 2.859	0.55	406	22.43 ± 2.995	0.16
	AT	60	26.43 ± 2.697		133	23.03 ± 3.387	
	AA	8	27.45 ± 2.534		8	22.75 ± 3.576	
rs9940128	GG	186	26.40 ± 2.829	0.61	355	22.41 ± 2.950	0.08
	AG	75	26.25 ± 2.840		178	23.00 ± 3.383	
	AA	14	27.07 ± 2.515		17	21.88 ± 3.160	

The analysis of the associations between the nine SNPs and the severity of NAFLD is reported in Table 4. Rs1477196 was associated with the severity of NAFLD, and carriers of the AA genotype showed approximately a 2.95-fold increased risk of the moderate–severe NAFLD, compared with the AG + GG carriers (OR = 2.95, 95% CI = 1.09–7.94, *P* = 0.034). The other SNPs were not related to the severity of NAFLD (Table 4).

**Table 4:** Distribution of the genotypes of *FTO* and severity of the disease studied in the NAFLD group

		Genotype frequencies, *N*			
		Mild	Moderate–severe	OR (95% CI)	*P*
rs1121980					
Dominant	(AA + AG)/GG	54/127	20/60	0.62 (0.35–1.12)	0.11
Recessive	AA/(AG + GG)	10/181	2/78	0.39 (0.09–1.79)	0.18
Additive	AA/AG/GG	10/44/127	2/18/60	0.64 (0.39–1.06)	0.071
rs1477196					
Dominant	(AA + AG)/GG	66/128	28/52	1.04 (0.60–1.80)	0.88
Recessive	AA/(AG + GG)	8/186	9/71	2.95 (1.09–7.94)	0.034
Additive	AA/AG/GG	8/58/128	9/19/52	1.24 (0.82–1.89)	0.32
rs17817449					
Dominant	(GG + TG)/TT	52/143	16/64	0.69 (0.37–1.29)	0.24
Recessive	GG/(TG + TT)	7/188	1/79	0.34 (0.04–2.81)	0.26
Additive	GG/GT/TT	7/45/143	1/15/64	0.69 (0.39–1.20)	0.17
rs7195539					
Dominant	(AG + GG)/AA	31/163	16/65	1.21 (0.61–2.40)	0.58
Recessive	GG/(AA + AG)	4/190	1/79	0.34 (0.04–2.81)	0.095
Additive	GG/GA/AA	4/27/163	1/15/64	1.04 (0.56–1.92)	0.90
rs8050136					
Dominant	(AA + AC)/CC	52/143	16/64	0.69 (0.37–1.29)	0.24
Recessive	AA/(AC + CC)	7/188	1/79	0.34 (0.04–2.81)	0.26
Additive	AA/AC/CC	7/45/143	1/15/64	0.69 (0.39–1.20)	0.17
rs8061518					
Dominant	(AG + GG)/AA	130/65	53/27	0.98 (0.57–1.70)	0.95
Recessive	GG/(AA + AG)	41/154	16/64	0.94 (0.49–1.79)	0.85
Additive	GG/AG/AA	41/89/65	16/37/27	0.97 (0.68–1.39)	0.88
rs9921255					
Dominant	(TC + CC)/TT	35/154	18/61	1.30 (0.68–2.47)	0.43
Recessive	CC/(TC + TT)	2/187	1/78	1.20 (0.11–13.41)	0.88
Additive	CC/TC/TT	2/33/154	1/17/61	1.26 (0.70–2.27)	0.45
rs9939609					
Dominant	(AA + TA)/TT	52/143	16/64	0.69 (0.37–1.29)	0.24
Recessive	AA/(TT + TA)	7/188	1/79	0.34 (0.04–2.81)	0.26
Additive	AA/AT/TT	7/45/143	1/15/64	0.69 (0.39–1.20)	0.17
rs9940128					
Dominant	(AA + AG)/GG	68/127	21/59	0.66 (0.37–1.19)	0.16
Recessive	AA/(GG + AG)	12/183	2/78	0.39 (0.09–1.79)	0.18
Additive	AA/AG/GG	12/56/127	2/19/59	0.67 (0.41–1.10)	0.10

## 4 Discussion

Although several risk factors for NAFLD [22,23] had already been determined, the discovery of new genetic markers will advance the identification of individuals susceptible to the development of this disease. This might help ease the burden of NAFLD on individuals and society through the use of screening and proper interventions in those at risk of developing NAFLD. Previous studies suggested that *FTO* gene polymorphisms were commonly correlated with metabolic disorders, especially central obesity, low-density lipoprotein (LDL), insulin resistance, and hypertriglyceridemia [24], which are tightly related to NAFLD [25]. Therefore, *FTO* gene polymorphisms might have important implications related to NAFLD.

In this study, we performed an association analysis of NAFLD with nine *FTO* gene polymorphisms that have been previously found to be associated with metabolic disorders. Our study found that *FTO* rs1477196 was significantly associated with NAFLD risk in a Chinese male population and carriers of the AA genotype increased the NAFLD risk, in comparison with AG + GG carriers. Besides, rs1477196 was also associated with the severity of NAFLD. Previous study [26] reported that rs1477196 A allele was associated with an increased risk of obesity that was closely related to NAFLD, which indirectly supported our results. Nevertheless, after further adjustments for BMI, the relationship of rs1477196 and NAFLD weakened, suggesting that the relationship might be partly dependent on BMI. We further evaluated the relationships of *FTO* gene polymorphisms with NAFLD risk stratified by BMI. Our results found that in obese men, rs1121980 and rs9940128 were associated with NAFLD risk in the dominant model after adjusting for BMI, uric acid, metabolic syndrome, smoking, and drinking. No significant correlations were observed between *FTO* gene polymorphisms and NAFLD risk when BMI < 25. These results indicated the interaction between *FTO* gene polymorphisms and obesity for NAFLD risk.

Moreover, an animal model study of *FTO* expression in rat liver with NAFLD proposed that overexpression of *FTO* enhances oxidative stress and lipid accumulation [27]. Oxidative stress is the core feature of the pathogenesis of NAFLD and plays a critical role in the progress of this disease [28]. Notwithstanding the mechanism of *FTO* gene on lipid overaccumulation in liver has not been previously studied, *FTO* overexpression increases the rate of lipogenesis [29]. Coincidentally, another study on fatty liver disease in HIV-infected patients also put forward a similar opinion [30]. They proposed that *FTO* gene variations might be independent predictors of fatty liver disease in HIV-infected patients. To a certain degree, the results that the *FTO* gene polymorphisms are associated with NAFLD risk in our study are in agreement with the literature and highlight the role of the gene in metabolic disorder in Chinese population.

There were some limitations in our study. First, only males were included in this study, so we should be prudent when extrapolating the findings to women, and studies on females should be considered in the future. Second, the sample size of this study is moderate, which limited the subsequent stratified analysis, such as the severity of NAFLD. Third, our study included nine SNPs and multiple testing might increase the false-positive (type I error) rate under nominal significance thresholds. Therefore, large population-based prospective studies are needed to elucidate the impact of *FTO* SNPs on NAFLD risk.

## 5 Conclusion

Our results demonstrated that the *FTO* gene was related to the presence and severity of NAFLD in a Chinese male population, and the relationships of the tested SNPs with NAFLD are most probably mediated by BMI. In order to better uncover the relationships between *FTO* gene polymorphisms and NAFLD, further investigations would be required to assess the clinical consequences of *FTO* affecting hepatic fatty infiltration in different races, particularly among those who are overweight or obese.
